# High number of seizures and unconsciousness in patients with SARS-CoV-2 omicron variants: a retrospective study

**DOI:** 10.3389/fped.2023.1273464

**Published:** 2023-11-15

**Authors:** Kishin Tokuyama, Tsubasa Kitamura, Kazutaka Maruyama, Shun Toriumi, Yayoi Murano, Daisuke Yoneoka, Tomoyuki Nakazawa, Toshiaki Shimizu

**Affiliations:** ^1^ Division of Pediatrics, Tokyo Metropolitan Toshima Hospital, Tokyo, Japan; ^2^ Department of Pediatrics, Faculty of Medicine, Juntendo University, Tokyo, Japan; ^3^ Center for Surveillance, Immunization, and Epidemiologic Research, National Center of Infectious Disease, Tokyo, Japan

**Keywords:** COVID-19, SARS-CoV-2, omicron variants, febrile seizure, seizure

## Abstract

Severe acute respiratory syndrome coronavirus 2 (SARS-CoV-2) omicron variants are now a pandemic. There are differences in clinical features in SARS-CoV-2 variants and we conducted this study to assess the clinical features of coronavirus disease (COVID-19) in children with SARS-CoV-2 omicron variants. The study included children with COVID-19 arrivedto Tokyo Metropolitan Toshima Hospital between January 2020 and October 2022. The clinical features of 214 children with SARS-CoV-2 non-omicron variants and 557 children with omicron variants were compared. In the SARS-CoV-2 omicron variant group, more patients had fever, sore throat, nausea and/or vomiting, and seizures and/or disorders of consciousness. In SARS-CoV-2 non-omicron variants, there was only one patient with seizure and/or unconsciousness whereas there were 92 children in omicron variants. Among these 92 patients, 46 (49%) were diagnosed with simple febrile seizures; 23 (25%), with complex febrile seizures; 10 (11%) with status epilepticus; and two (2%) with encephalopathy. Their mean age was 4.0 ± 3.0 years—a wider age distribution than that in other febrile seizures but similar to that in febrile seizures in patients with influenza. SARS-CoV-2 omicron variants are likely to cause seizures and unconsciousness in children and their age distribution was wider than other febrile seizures patients but similar to those in influenza patients. In clinical practice in patients with COVID-19 and influenza, clinicians should be aware of these features.

## 1. Introduction

Coronavirus disease (COVID-19) caused a pandemic in 2019 and gradually becoming endemic. Since the beginning of the pandemic, children have been reported to experience milder symptoms than adults (1). However, the symptoms differ between variants (2), and the severe acute respiratory syndrome coronavirus 2 (SARS-CoV-2) omicron variant, which is one of the “variants of concern,” appeared at the end of 2021 with high transmissibility. These variants have been reported to affect the younger population (3), and the pandemic is said to be an urgent matter (4). Also, some studies have reported seizures in children with COVID-19 infected with omicron variants (5–8); however, most are case reports. Moreover, although there are several studies from Japan comparing omicron variant mainly include adult participants (9–12), little are known in children.

Therefore, this study aimed to reveal the clinical features of the SARS-CoV-2 omicron variants among children.

## 2. Methods

The study included children aged 0–15 years who were diagnosed as COVID-19 by SARS-CoV-2 PCR test or antigen test and treated for COVID-19 at Tokyo Metropolitan Toshima Hospital between January 2020 and October 2022. Data were electronically extracted from their medical records. The data included age at arrival at our hospital, date of arrival at our hospital, sex, and symptoms (fever, cough, rhinorrhea, headache, sore throat, nausea, vomiting, diarrhea, abdominal pain, rash, taste and smelling disorder, joint pain, seizures, disorders of consciousness, and abnormal urinalysis results). Our hospital is a designated medical institution for infectious disease and accepted patients with COVID-19 from very first time of pandemic in Japan. We accepted all patients with COVID-19, and also there were admission to our hospital instructed by health care center during the early phase of pandemic. We collected additional detailed information on seizures and unconsciousness, which included the type of seizures and the final diagnosis of seizures and unconsciousness. The seizure occurred once and stopped within five minutes was defined as simple febrile seizure. Seizure occurred more than twice within 24 h was defined as complex seizure, and seizure continuing when arriving at the hospital was defined as status epilepticus.

At the beginning of study period, COVID-19 was classified as a Category 2 disease under the Infectious Disease Control Law, but in February 2021, it was classified as a new type of influenza and other infectious diseases.

The study participants were divided into two groups. The first group comprised children who visited our hospital between January 2020 and December 2021—the period corresponding to when omicron was not the dominant variant in Japan (non-omicron group). The second group comprised children who visited our hospital from January 2022, when omicron was the dominant variant in Japan (omicron group) (13).

To analyze the basic characteristics of the participants, we performed a chi-square test for categorical variables and a *t*-test for continuous variables. Using the *χ*^2^ test, we compared the clinical features between the non-omicron and omicron group. Unknown values were treated as missing values.

Statistical analyses were performed using Stata, version 15.1 (Stata Corp., College Station, TX, USA). Statistical significance was set at *P* < 0.05.

This study was approved by the ethical committee of Tokyo Metropolitan Toshima Hospital (Approve number: Jin 2–32). The requirement for patient consent was waived owing to the use of anonymous data.

## 3. Results

During the study period, January 2020 to October 2022, there were 214 participants with non-omicron variant COVID-19 and 557 with an omicron variant.

Table 1 presents the participants' basic characteristics. There were 128 boys (59.8%) in the non-omicron group and 322 (57.8%) in the omicron group—the difference was not significant (*p* = 0.61). The mean [± standard deviation (SD)] age was 6.1 ± 4.7 years in the non-omicron group and 5.3 ± 4.4 years in the omicron group—the participants in the omicron group were significantly younger (*p* < 0.01). In the non-omicron and omicron group, 163 (76.2%) and 549 (98.6%) children had at least one symptom—this difference was significant (*p* < 0.001).

**Table 1:** Comparison of basic characteristics and symptoms of the participants between the non-omicron and omicron group.

	Non-omicron group (*n* = 214)	Omicron group (*n* = 557)	*p*-value
Sex, boys (%)^a^	128 (59.8%)	322 (57.8%)	0.61
Age, years (mean ± SD)^b^	6.1 ± 4.7	5.3 ± 4.4	<0.01
Number of patients with symptoms^a^	163 (76.2%)	549 (98.6%)	<0.001
Symptom^a^			
Fever	133 (62.1%)	534 (95.9%)	<0.001
Cough, rhinorrhea	87 (40.7%)	177 (31.8%)	0.02
Headache^c^	20 (19.2%)	68 (27.4%)	<0.001
Taste/Smelling disorder^c^	13 (17.6%)	3 (2.1%)	<0.001
Sore throat^c^	13 (9.1%)	70 (20.3%)	<0.001
Nausea/vomiting	14 (6.5%)	103 (18.5%)	<0.001
Diarrhea/abdominal pain	9 (4.2%)	36 (6.4%)	0.23
Rash	5 (2.3%)	7 (1.2%)	0.27
Joint pain^c^	2 (1.5%)	8 (2.6%)	<0.001
Seizures/disorders of consciousness	1 (0.5%)	92 (16.5%)	<0.001
Abnormal urine analysis	1 (0.5%)	1 (0.02%)	0.48

SD, standard deviation.

^a^ Calculated using the *χ*^2^ test.

^b^ Calculated using the *t*-test.

^c^ Those who cannot tell the symptom are excluded.

Table 1 also presents the results of the comparison between the two groups stratified according to the participants' symptoms. The number of children with fever, headache, taste/smelling disorder, sore throat, nausea and/or vomiting, joint pain, and seizures and/or disorders of consciousness was significantly higher in the omicron group. In contrast, the number of patients with taste and/or smelling disorders was significantly higher in the non-omicron group. Other symptoms, including cough and/or rhinorrhea, diarrhea and/or abdominal pain, rash, and abnormal urinalysis results, were not significantly different between the groups.

**Figure 1:**
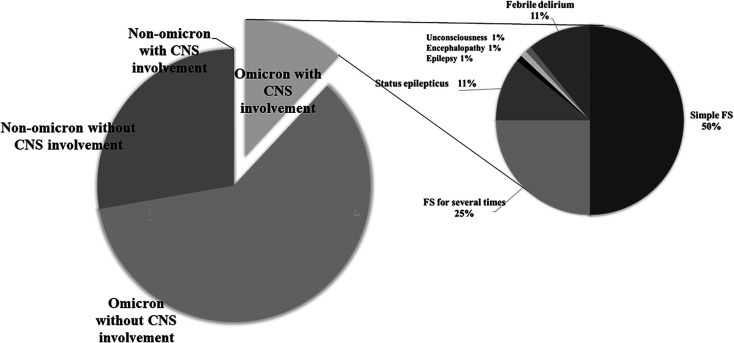
Final diagnoses of patients with seizures and unconsciousness.

Figure 1 shows the final diagnosis of patients with seizures and unconsciousness. Among the 92 children, 46 (50%) were diagnosed with simple febrile seizures (FS); 23 (25%), who had several seizures, with complex FS; 10 (11%) with status epilepticus, and two (2%) with encephalopathy. Of the remaining children, 10 were diagnosed with febrile delirium, and one with epilepsy. Their mean age was 4.0 ± 3.0 years (lowest: 2 months, highest: 11 years). There were 64 boys (69.6%).

Finally, the comparison of the two groups in presence of seizure or unconsciousness among children with fever and there was significant difference (*p* < 0.001): *Among 133 febrile participants in non-omicron group, one had the symptom, whereas among 534 febrile participants in omicron group 92 had the symptom*. The comparison of presence of simple or complex FS between the two groups also showed significant difference (*p* < 0.001): *Among 133 febrile participants in non-omicron group, one had the symptom, whereas among 534 febrile participants in omicron group 69 had the symptom. All participants with seizure or unconsciousness were discharged without any complications*.

## 4. Discussion

In this study, we aimed to reveal the clinical features of the SARS-CoV-2 omicron variants among children, and revealed that, in comparison with other SARS-CoV-2 variants, the omicron variant was associated with more clinical symptoms, particularly seizures. The number of children with seizures and unconsciousness was significantly higher after than before the emergence of the SARS-CoV-2 omicron variant.

We considered the SARS-CoV-2 pandemic in terms of variants, where the non-omicron period extended over 24 months, from January 2020 to December 2022, and the omicron period extended over 10 months, from January 2022 to October 2022. Despite the omicron period having been shorter, there were more children with COVID-19 than there were during the non-omicron period. This result is consistent with that of one study that reported more children having been admitted during the SARS-CoV-2 omicron period (14) and another that reported an increased admission rate of children under one year (15). Based on the results of the present and previous studies, it can be concluded that SARS-CoV-2 omicron variants affect more children than do previous variants. Moreover, the lower mean age of the SARS-CoV-2 omicron group in our study supports these results.

We emphasize that there were many more children who had seizures and unconsciousness during the SARS-CoV-2 omicron period, because there was only one patient in the non-omicron group and 92 in the omicron group. In addition, the age of children with seizures and unconsciousness is noteworthy. FS are likely to occur between the ages of 6 months and 6 years (16). However, in our study sample, the age range was wider, and both younger and older patients had seizures and unconsciousness. This finding is similar to that for influenza, which has a higher tendency to cause FS in older age than do other diseases (17). During the COVID-19 pandemic, influenza has also started to cause an epidemic (18, 19) and clinicians should be aware of this tendency in their clinical practice.

Some studies have reported seizures in children with COVID-19 infected with omicron variants (5–8); however, most are case reports. In the present study, we statistically analyzed and determined the association of seizures and unconsciousness with SARS-CoV-2 omicron variants. Although the pathology is not well known, neurological manifestations of SARS-CoV-2 omicron variants in the adult population have been reported (20).

Our study has some limitations. First, we considered the SARS-CoV-2 pandemic in terms of variants; however, we did not confirm the variants with laboratory diagnostic tools. Nevertheless, the period that we defined as the SARS-CoV-2 pandemic is reported to be a pandemic of more than 90% omicron variants. Also, due to strict indication of neuroimage because of infection control, we could not diagnose encephalopathy and some participants may be categorized se status epilepticus. However, their clinical symptom do not require further investigation after isolation period and they had good prognosis. Second, patients' diagnostic thresholds have been changing. During the very early term of the pandemic, children in close contact were carefully screened, and more asymptomatic patients with COVID-19 were diagnosed compared to recently. As children are more likely to be asymptomatic (1), we cannot avoid selection bias in the study sample. On the other hand, there is a possibility that people came to hospital less frequently in the early phase of pandemic and the number of COVID-19 children are estimated lower. However, parents bring their children if they have seizure or unconsciousness, our conclusion still have impact on further clinical practice. Third, we could not obtain information about vaccination status. However, as they were introduced to Japanese children in later phase of pandemic with omicron, it can be said that even vaccination was done, there is still larger number of seizure in omicron variant infected children.

## 5. Conclusion

In conclusion, our study revealed that SARS-CoV-2 omicron variants are associated with increase in seizure and unconsciousness in children with COVID-19. As this study sample was drawn from a single institution, further studies including more institutions and larger sample sizes are required.

## Data Availability

The raw data supporting the conclusions of this article will be made available by the authors, without undue reservation.
