# Supplementary material for: High number of seizures and unconsciousness in patients with SARS-CoV-2 omicron variants: a retrospective study
Source: Front Pediatr. 2023 Nov 15;11:1273464. doi: 10.3389/fped.2023.1273464 (PMC10684743; doi:10.3389/fped.2023.1273464)
Supplement: Supplementary file 1 [file Table1.pdf]

Supplemental table 1

Symptom		
	Non-omicron group (n=214)	Omicron group (n=557)
	Yes/ No/ Data not available	Yes/ No/ Data not available
Fever	133/81/0	534/23/0
Cough, rhinorrhea	87/127/0	177/380/0
Headache	20/84/110	68/180/309
Taste/ Smelling disorder	13/61/140	3/140/414
Sore throat	13/130/71	70/275/212
Nausea/ vomiting	14/200/0	103/454/0
Diarrhea/ abdominal pain	9/205/0	36/521/0
Rash	5/209/0	7/550/0
Joint pain	2/130/82	8/300/249
Seizures/disorders of consciousness	1/213/0	92/465/0
Abnormal urine analysis	1/213/0	1/557/0
